# FOXO1 Overexpression Attenuates Tubulointerstitial Fibrosis and Apoptosis in Diabetic Kidneys by Ameliorating Oxidative Injury via TXNIP-TRX

**DOI:** 10.1155/2019/3286928

**Published:** 2019-03-06

**Authors:** Linlin Ji, Qingzhu Wang, Fengjuan Huang, Tingting An, Feng Guo, Yanyan Zhao, Yang Liu, Yanyan He, Yi Song, Guijun Qin

**Affiliations:** ^1^Department of Endocrinology and Metabolism, First Affiliated Hospital of Zhengzhou University, Zhengzhou, 450052 Henan Province, China; ^2^Institute of Clinical Medicine, First Affiliated Hospital of Zhengzhou University, Zhengzhou, 450052 Henan Province, China

## Abstract

**Objective:**

The generation of hyperglycemia-induced reactive oxygen species (ROS) is a key event in diabetic nephropathy (DN) development. Since forkhead box class O1 (FOXO1) is associated with oxidative stress and shows a positive effect on DN, its role on renal function and the underlying mechanism is still unclear.

**Methods:**

We examined the role of FOXO1 *in vivo* (in a transgenic diabetic mouse model overexpressing *Foxo1*) and *in vitro* (in human HK-2 cells with *FOXO1* knockin (KI) and knockout (KO) cultured under high glucose).

**Results:**

Renal proximal tubular cells of kidney biopsies from patients with DN showed tubulointerstitial fibrosis and apoptosis. Accordingly, these proximal tubular injuries were accompanied by the increase of ROS generation in diabetic mice. Tissue-specific *Foxo1* overexpression in transgenic mice had a protective effect on the renal function and partially reversed tubular injuries by attenuating the diabetes-induced increase in TXNIP and decrease in the TRX levels. *FOXO1* knockin and knockout HK-2 cells were constructed to identify the associations between FoxO1 and TXNIP-TRX using CRISPR/CAS9. Similarly, the effects of *FOXO1* KI and KO under high glucose were significantly modulated by the treatment of TRX inhibitor PX-12 and *TXNIP* small interfering RNA. In addition, *TXNIP* and *TXN* were identified as the direct FOXO1 transcriptional targets by chromatin immunoprecipitation.

**Conclusion:**

The regulatory role of FOXO1/TXNIP-TRX activation in DN can protect against the high glucose-induced renal proximal tubular cell injury by attenuating cellular ROS production. Modulating the FOXO1/TXNIP-TRX pathway may be a new therapeutic target in DN.

## 1. Introduction

Reactive oxygen species (ROS) are increasingly recognized as the most important factors regulating altered metabolic pathways in differentiated cells, ultimate contributing to inflammation, fibrosis, dysfunction, and apoptosis [1, 2]. Diabetic nephropathy (DN) is an important cause of end-stage renal disease [3], and accumulating evidence implicates renal proximal tubular cells (RPTCs) as drivers of the structural and functional changes in diabetic kidneys [4, 5]. Given that ROS generation in RPTCs is increased under diabetic conditions [6], ROS accumulation might accelerate the irreparable progression of DN.

Thioredoxin-interacting protein (TXNIP) is a negative regulator of thioredoxin (TRX). TXNIP-TRX appear to be important contributors among the enzymatic systems implicated in ROS generation and renal oxidative stress [7]. Chronic exposure of hyperglycemia increases the production of ROS, resulting in the serious damage by promoting DNA damage, lipid peroxidation, and protein modification [8]. ROS also plays a critical role in stimulating the growth factors and cytokines like transforming growth factor-beta 1 and connective tissue growth factor [9]. These ROS-induced profibrotic factors lead to an excessive buildup of extracellular matrix, which further exacerbates kidney injury [10].

Forkhead transcription factor O1 (FOXO1) is a member of the forkhead box-containing transcription factor O family. Activation of the PI3K/Akt pathway under a high-glucose condition phosphorylates three threonine sites of FOXO1 and promotes its translocation from the nucleus to the cytoplasm, resulting in the loss of its function [11]. FOXOs have been reported to function together with TXNIP [12] and TRX [13] to mediate oxidative stress in pancreatic *β* cells and endothelial cells. However, the understanding of the involvement of FOXO1 in TXNIP-TRX mediated ROS accumulation in RPTCs, and their relationship are still limited.

Therefore, the present study aims at elucidating how RPTCs respond to diabetes-induced ROS accumulation under the condition of FOXO1 overexpression, contributing to interstitial fibrosis and apoptosis, and at determining whether the effects occur downstream of the TXNIP-TRX. Toward this end, we compared the RPTC damage in kidney biopsy samples from patients with and without DN. To determine the role of FOXO1 in this damage and the underlying mechanisms, we established a tissue-specific transgenic (Tg) diabetic mouse model overexpressing *Foxo1* and determined the effects on physiological parameters, extent of apoptosis and interstitial fibrosis, and production of ROS. Moreover, we examined the interaction between FOXO1 and TXNIP-TRX in mediating these effects using high glucose-treated human RPTCs *in vitro*. These results should contribute new insight into the molecular mechanisms underlying the development of DN and highlight potential therapeutic targets for treatment and prevention.

## 2. Materials and Methods

### 2.1. Kidney Biopsy Specimens

Ten human subjects with suspected kidney pathology underwent a standard punch kidney biopsy at the First Affiliated Hospital of Zhengzhou University. Five patients were diagnosed with type 2 diabetes for more than 10 years accompanied by microalbuminuria (30-300 mg/g Cre) as well as retinopathy. The other five individuals were classified as normal glucose-tolerant accompanied by IgA nephropathy with minimal proteinuria.

All patients provided written informed consent in human studies, and the protocols for experiments with human tissues were approved by the scientific-ethical committees of the First Affiliated Hospital of Zhengzhou University and adhered to the Declaration of Helsinki guidelines.

### 2.2. Generation of CA-Foxo1 Tg Mice

The CA-*Foxo1* construct included a specific cDNA fragment encoding full-length *Foxo1* with mutated positions in Thr24, Ser256, and Ser319 to prevent phosphorylation when fused with the GFP-tag. Tg mice (C57BL/6 background) overexpressing CA-*Foxo1* in the kidney were established by embryo microinjection at ViewSolid Biotech (Beijing, China) and then crossed with wild-type mice (C57BL/6 background) purchased from Charles River (Beijing, China). CA-*Foxo1* Tg mice were identified by PCR amplification of the genomic DNA sequence for the transgene using the following primers: CA-*Foxo1* sense primer 5′- TTACAAGTGCCTCTGTCCCACC-3′ and the GFP (a tag sequence encoding amino acid residues) antisense primer 5′- ACTTCAGGGTCAGCTTGCCGTAG-3′.

### 2.3. Animal Experimental Protocol

Nine Tg male mice and their wild-type littermates at the age of 8 weeks were used in the present study. All animals received standard mouse diet and water *ad libitum*. The Animal Care and Use Committee of Zhengzhou University approved all animal experiments. After 12 h of fasting, diabetes was induced in wild-type and Tg mice by one intraperitoneal injection of a 130 mg/kg streptozotocin (Sigma; St. Louis, MO, USA) solution in 0.05 M citrate buffer (pH 4.5), serving as the diabetes group and *Foxo1* Tg diabetes group. Wild-type mice injected with citrate buffer served as the normal control group. Blood glucose was measured to confirm the successful establishment of the diabetes model at 72 h after streptozotocin injection, which was defined as a blood glucose level higher than 16.7 mmol/L. Body weight was recorded and blood glucose was monitored every 2 weeks for a period of 12 weeks as described in our previous study [14]. A 24 h urine sample was collected to determined 24 h UTP, 8-OHDG, and creatinine levels 12 weeks after injection. The mice were sacrificed with chloral hydrate, and blood was collected form the orbital vein to measure Scr and urea. The kidney weight/body weight ratios were subsequently determined. Kidney proximal tubules were collected using Precoll as described previously [15]. Separated cortexes were fixed in either 4% paraformaldehyde for staining or 4% precooled glutaraldehyde for transmission electron microscopy. The remaining kidney cortex was stored in liquid nitrogen for subsequent experiments.

### 2.4. Establishment of FOXO1 Knockin (KI) and Knockout (KO) Cell Lines through CRISPR/Cas9

*FOXO1* KI and KO HK-2 cell lines were established using the CRISPR/Cas9 system according to a standard protocol. The AAVS1 safe harbor gene knockin kit was provided by GeneCopoeia (Rockville, MD, USA), and the donor plasmid was a constitutively active form of *FOXO1* with mutated positions in Thr24, Ser256, and Ser319 (NM_002015.3). The two plasmids were cotransfected into HK-2 cells for 24 h by EndoFectin Max (GeneCopoeia). Subsequently, the cells were selected by puromycin for another 24 h to eliminate nontransfected cells. DNA was extracted for the verification of the recombination sites by PCR. Successfully edited HK-2 cells were then seeded onto 96-well plates for monoclonalization. FOXO1 expression of cell clones was detected by western blotting and sequencing. HK-2 cells were transfected with the all-in-one sgRNA clone for the human *FOXO1* gene (HCP205747-CG01-3-10a, c; GeneCopoeia) by EndoFectin Max for 72 h. The target sites of HCP205747-CG01-3-10a and c were GGACTGGCTAAACTCCGGCC and GGCTGCCAACCCCGACGCCG, respectively. After that, the cells were selected by G418 for another 48 h. DNA was extracted for screening using the T7 endonuclease I assay kit (GeneCopoeia). Subsequently, limiting dilution was performed to isolate single cells. Single clones were allowed to grow for about 3 weeks prior to testing via immunoblotting and sequencing to identify *FOXO1* KO clones. Cells with successful genotyping were used for subsequent experiments.

### 2.5. Cell Culture and Treatments

HK-2 cells (American Type Culture Collection, Manassas, VA, USA) were cultured in Dulbecco's modified Eagle's medium (low glucose, 1 g/L) (Gibco, Grand Island, NY, USA) supplemented with 10% fetal bovine serum (Gibco, Grand Island, NY, USA) and 1% penicillin-streptomycin solution at 37°C in a 5% CO_2_ atmosphere. The TRX1 inhibitor PX-12 was purchased from SelleckChem and used at a final working concentration of 10 *μ*m for 16 h. *TXNIP* expression was silenced in *FOXO1* KO cells using siRNA specifically targeting *TXNIP* (RiboBio Co., Guangzhou, China). 24 h prior to transfection, *FOXO1* KO cells were plated into a 6-well plate. Then cells were transfected with 50 nM si-*TXNIP* or siNC (negative control) by EndoFectin Max according to the manufacturer's protocol for 4-6 h. The medium was replaced with fresh culture medium after treatments, and cells were harvested for stimulation in high glucose (HG, 4.5 g/L) at 48 h.

### 2.6. Quantitative Real-Time PCR

Total RNA was extracted from RPTCs and cultured HK-2 cells using TRIzol reagent (TaKaRa Bio, Shiga, Japan). cDNA was synthesized from RNA, and quantitative PCR was performed as described previously [10]. The primer sequences used for PCR are shown in Table 1. The reactions were performed on an ABI Fast 7500 cycler (Applied Biosystems, Foster City, CA, USA). Relative expression standardized to *GAPDH* was calculated using the comparative cycle threshold method (2^-ΔΔCt^).

### 2.7. Western Blot Analysis

After different treatments, total protein from HK-2 cells and RPTCs was extracted and western blotting was performed as described previously [14] using the following primary antibodies: rabbit anti-FOXO1 and TXNIP (Abcam, Cambridge, UK); rabbit anti-p-FOXO1 Ser256 (Cell Signaling Technology, Danvers, MA, USA); rabbit anti-TRX, anti-FN, anti-Col IV, anti-BAX (Proteintech, Chicago, IL, USA), and anti-*β*-actin (Sangon Biotech, Shanghai, China). Signals were visualized in the samples by enhanced chemiluminescent substrate (Life Technology) after incubation with a horseradish peroxidase-conjugated anti-rabbit secondary antibody (Sangon Biotech). The relative optical density of each band was quantitated using ImageJ software (National Institutes of Health, Bethesda, MD, USA), and *β*-actin was probed to ensure equal protein loading.

### 2.8. Immunohistochemistry Analysis

Paraffin sections of the mouse kidneys were prepared by a conventional method and treated with the immunohistochemistry staining protocol as described previously [14]. The working concentrations of the FOXO1 and TXNIP antibodies were 1 : 100 and 1 : 25, respectively. The distribution and subcellular localization of the target proteins were examined by light microscopy (Olympus, Tokyo, Japan). Densitometric analysis was performed using Image-Pro Plus software version 6.0 (Media Cybernetics, Rockville, MD, USA).

### 2.9. ChIP Assays

ChIP assays were conducted using the EZ-ChIP Kit (Millipore, Billerica, MA, USA) according to the manufacturer's instructions as described previously [14]. De-cross-linked DNA samples were subjected to PCR amplification using forward (5′- AGCGCAACAACCATTTTCCC-3′) and reverse (5′- TTGTTTACCAGGAGCCCGAC-3′) primers targeting the *TXNIP* promoter and forward (5′- GCGTGCTGTGCCATTGTAAA-3′) and reverse (5′- CTTGCAAAGGACGGTGCTTG-3′) primers targeting the *TRX* promoter. Precipitated DNA fragments were analyzed by quantitative PCR.

### 2.10. Measurement of Intracellular ROS Accumulation

The intracellular ROS accumulation was detected using the 2′7-dichlorodihydrofluorescein-diacetate (DCFH-DA) probe (Beyotime, Shanghai, China) by flow cytometry as described previously [16]. In brief, after treatments for the indicated time intervals, cells were harvested and washed twice with phosphate-buffered saline (PBS), incubated with DCFH-DA at a final concentration of 10 mmol/L in PBS at 37°C for 20 min, washed twice with PBS, and analyzed by flow cytometry. Fluorescence intensity was analyzed by flow cytometry using excitation/emission wavelengths of 488/525 nm.

### 2.11. Measurement of 8-OHDG and MDA

Urinary samples were centrifuged at 4000 rpm, and then each sample was assessed for 8-OHDG using an enzyme-linked immunosorbent assay kit from Cusabio Biotech. All the reagents were of analytical grade. Concentrations of 8-OHDG were estimated by a spectrophotometer and calculated by measuring the optical density of 575 nm. Renal cortex MDA levels were quantified using the lipid peroxidation MDA assay kit (Beyotime Institute of Biotechnology, Jiangsu, China), according to the manufacturer's protocol and as described in our previous report [16].

### 2.12. Apoptosis Assay

A TdT-mediated dUTP nick-end labeling (TUNEL) staining kit (Roche, Basel, Switzerland) was used to detect apoptosis according to the manufacturer's instructions. Numbers of TUNEL-positive tubular cells were quantified by counting 10 randomly chosen nonoverlapping fields per slide. All slides were observed independently by two blinded investigators.

Treated cells were washed with PBS, resuspended in binding buffer, and stained with annexin V-fluorescein isothiocyanate (FITC) and propidium iodide (PI) for 15 min according to the kit protocol from BD Biosciences. Annexin V-FITC and PI fluorescence were estimated by the use of a flow cytometer (Beckman), according to the manufacturer's instructions.

### 2.13. Light Microcopy and Electron Microscopy

Kidney pathology in hematoxylin and eosin, periodic acid-Schiff, and Masson trichrome sections was examined by light microscopy. Renal cortexes were sectioned into 1 mm^3^ and 1 cm^3^ cubes with a cold blade and fixed with 4% glutaraldehyde. The ultrastructure of the renal cortex was observed with a transmission electron microscope (H-7500; Hitachi, Tokyo, Japan). The investigators were blinded to the treatment conditions when performing the electron microscopy observations.

### 2.14. Statistical Analysis

The data were analyzed using SPSS 17.0 software (IBM SPSS, Watson, NY, USA) and are expressed as the mean ± standard error of the mean. The differences among the experimental groups were assessed using the one-way analysis of variance followed by the Bonferroni test for multiple comparisons and the multiple range test. *P* value < 0.05 was considered statistically significant.

## 3. Result

### 3.1. DN Kidneys Show Signs of Nuclear Damage and Apoptosis

**Figure 1:**
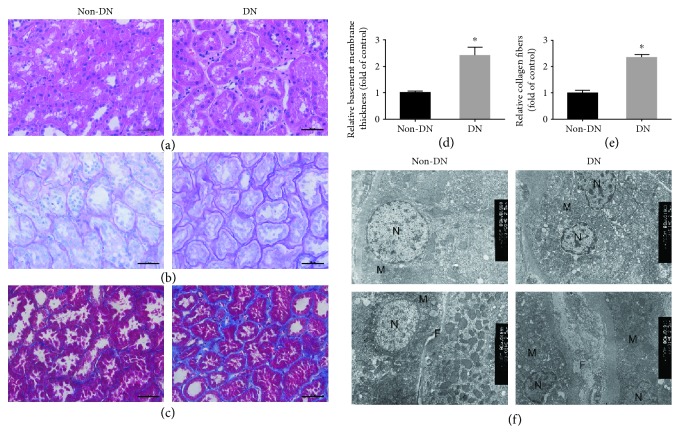
Renal proximal tubular cell (RPTC) injury in patients with diabetic nephropathy (DN). (a) Representative images of hematoxylin and eosin staining, (b) periodic-acid Schiff staining, and (c) Masson's trichrome staining in kidney biopsy specimens from patients with DN and non-DN. Scale bars, 50 *μ*m. (d) Relative basement membrane thickness in PAS staining. (e) Relative collagen fibers in Masson staining. (f) Nucleus (N), mitochondria (M) of RPTCs, and interstitial collagen fibers (F) as viewed by transmission electron microscopy. Original magnification, 4000x. All data are expressed as means ± SEM, *n* = 6 (^∗^*P* < 0.05 vs. the non-DN).

To examine the extent of RPTC damage in DN, kidney biopsy samples taken from patients with and without DN were analyzed. Atrophy of proximal tubular cells and thickening of the tubular basement membrane were observed in the kidneys of patient with DN based on hematoxylin and eosin and periodic-acid Schiff staining (Figures 1(a), 1(b), and 1(d)). Likewise, Masson's trichrome staining indicated enhanced expression of collagen compounds in DN kidneys (Figures 1(c) and 1(e)).

Transmission electron microscopy showed that the RPTCs of non-DN patients had normal round nucleus with no apoptotic morphology and normal collagen fibers, whereas those of patients with DN showed morphological features of apoptosis. Moreover, a reduction in volume and formation of membrane protrusion were observed in the nucleus, along with the reorganization of chromatin aggregation and edge accumulation, potentially indicating the early stages of nuclear envelope rupture. Substantial interstitial collagen fibers were also detected in the kidneys from DN patients, resulting in a thickened tubular basement membrane (Figure 1(f)).

### 3.2. Successful Generation of Tg Mice with Kidney-Specific *Foxo1* Expression

**Figure 2:**
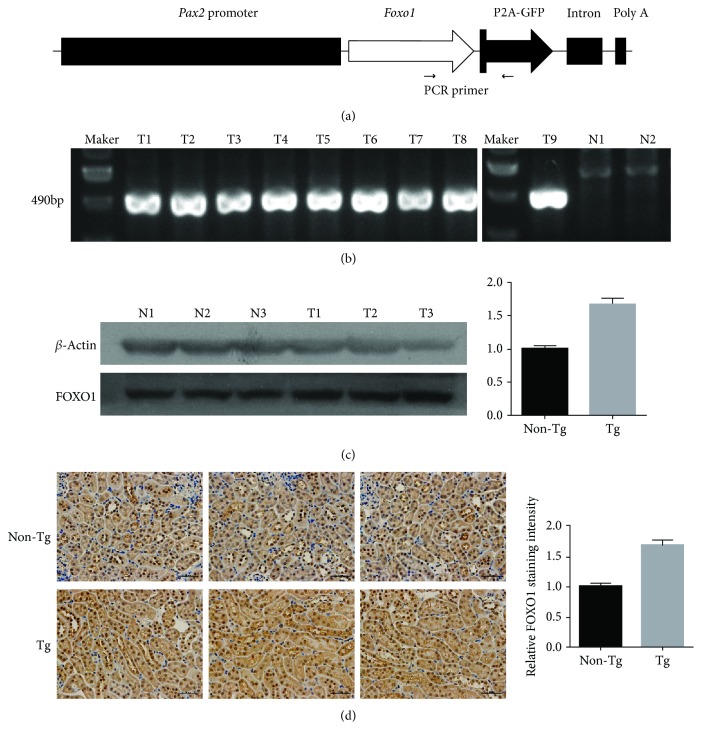
Tissue-specific expression of the *Pax2*-*Foxo1* transgene in transgenic (Tg) mice. (a) Piggy-*Pax2-Foxo1* construct for the generation of Tg mice. Schematic representation of the construct containing the *Pax2* promoter, CA*-Foxo1*, and GFP sequences. The positions of PCR primers used to detect the transgene are shown. (b) Genomic identification of Tg mice. *Foxo1*-Tg mice (T1–T9) showed a PCR-amplified 490 bp product that was undetected in non-Tg mice (N1–N2). (c) Western blot analysis of FOXO1 expression in mouse renal proximal tubule extracts of male non-Tg (N1-N3) and Tg (T1-T3) mice; *β*-actin was used as an internal control. (d) Immunohistochemistry staining of FOXO1 and relative staining intensity in male non-Tg and Tg mouse kidneys, using rabbit anti-FOXO1 polyclonal antibodies. Scale bars, 50 *μ*m.

Tg mice were generated to produce specific and inducible expression of constitutively active- (CA-) *Foxo1* in the kidney using a *Pax2-Foxo1* construct (Figure 2(a)). Expression of the green fluorescent protein- (GFP-) tagged CA-*Foxo1*-GFP transgene directed by *Pax2* was confirmed in the progeny of the CA-*Foxo1* line 381 crossbred with C57BL/6 mice using polymerase chain reaction (PCR) (Figure 2(b)). Animals displaying the 490-bp *Foxo1*-GFP fragment were used in subsequent experiments. Western blot analysis further confirmed that FOXO1 protein expression was increased in the RPTCs of *Foxo1*-Tg mice compared with that of the wild type (Figure 2(c)), and enhanced FOXO1 expression was detected in the RPTCs of *Foxo1*-Tg mice by immunohistochemistry (Figure 2(d)). Taken together, these results demonstrated that *Pax2* directs CA-*Foxo1* transgene expression in the RPTCs of *Foxo1*-Tg mice.

### 3.3. *Foxo1* Overexpression Has a Protective Effect in Diabetic Mice

The body weights of male wild-type diabetic and Tg diabetic mice were significantly lower, whereas the blood glucose levels were significantly higher than those of normal mice. There was no significant difference in the body weight and blood glucose level between diabetic and Tg diabetic mice. These results indicate that *Foxo1* overexpression in the kidney alone is ineffective in preventing weight loss and hyperglycemia in diabetic mice.

Prominent increases in the kidney weight/body weight ratio, 24 h urinary total protein (UTP), serum creatinine (Scr), and urea levels were detectable in both diabetic and Tg diabetic mice after week 12 compared with those of normal mice. However, these parameters were significantly reduced in Tg diabetic mice compared with diabetic mice, demonstrating that *Foxo1* overexpression had a protective effect on the renal function in the diabetic mice (Table 2).

### 3.4. *Foxo1* Overexpression Prevents Interstitial Fibrosis and Apoptosis in the RPTCs of Diabetes Mice

**Figure 3:**
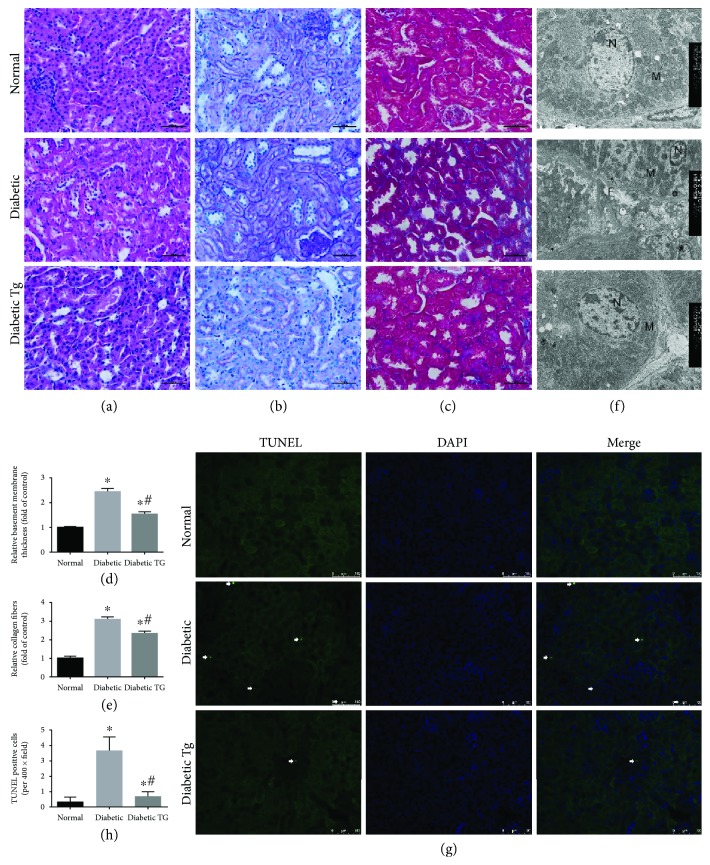
Histological changes of male transgenic (Tg) and non-Tg mouse kidneys at week 12. (a) Hematoxylin and eosin staining, (b) periodic-acid Schiff staining, and (c) Masson's trichrome staining of normal, diabetic, and diabetic transgenic (Tg) mouse kidneys at week 12. Scale bars, 50 *μ*m. (d) Relative basement membrane thickness in PAS staining. (e) Relative collagen fibers in Masson staining. (f) Nucleus (N), mitochondria (M) of RPTCs, and interstitial collagen fibers (F) as viewed by transmission electron microscopy. Original magnification, 4000x. (g) Representative pictures of TUNEL staining. Scale bars, 100 *μ*m. (h) Quantitative analysis of the number of apoptotic cells per field. All data are expressed as means ± SEM, *n* = 6 (^∗^*P* < 0.05 vs. the normal group and ^#^*P* < 0.05 vs. the diabetes group).

We next examined the effect of *Foxo1* overexpression on the morphological change characteristic of interstitial fibrosis and apoptosis. Hematoxylin and eosin and periodic-acid Schiff staining revealed the atrophy of proximal tubular cells and thickening of the tubular basement membrane in the kidneys of diabetic mice, whereas the control mice showed a normal kidney morphology. However, these changes were attenuated in *Foxo1* Tg diabetic mice (Figures 3(a), 3(b), and 3(d)). Likewise, Masson's trichrome staining showed enhanced expression of collagen in the kidneys of diabetic mice compared with that of the normal mice, although collagen components were normalized in the Tg diabetic mouse kidneys (Figures 3(c) and 3(e)). Similarly, transmission electron microscopy revealed a significant apoptosis in RPTCs and a substantial amount of interstitial collagen fibers in diabetic mice. In contrast, *Foxo1* overexpression prevents these injuries to some extent, indicating a protective effect (Figure 3(f)). We also performed TUNEL assays on the kidney sections to determine apoptosis. In comparison to normal mice, RPTCs from diabetic mice resulted in elevated apoptosis, and *Foxo1* overexpression could significantly decrease tubular cell apoptosis in diabetic mice (Figures 3(g) and 3(h)).

**Figure 4:**
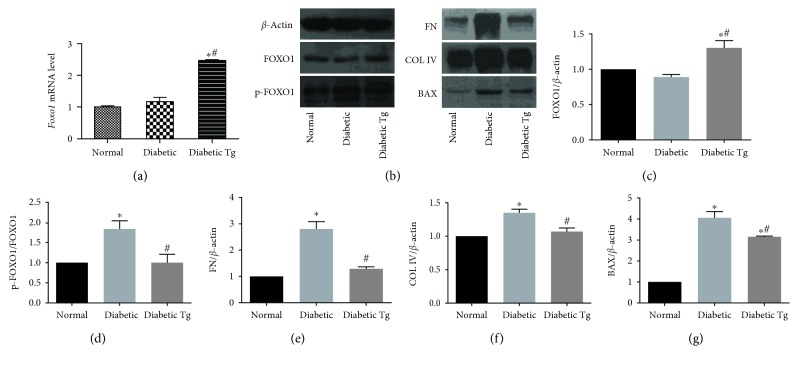
Effect of FOXO1 on apoptosis and interstitial fibrosis in the kidneys from diabetic mice. (a) *Foxo1* mRNA levels in renal proximal tubules of normal, diabetic, and diabetic Tg mice detected by qPCR at week 12; *Actb* was used as an internal control. (b) Representative immunoblots. (c, d) Protein levels of the total FOXO1 and p-FOXO1 detected by western blot analysis at week 12; *β*-actin was used as an internal control. (e–g) *Foxo1* overexpression prevented the increase in the expression of fibronectin (FN), collagen IV (Col IV), and BAX proteins. All data are expressed as means ± SEM, *n* = 6 (^∗^*P* < 0.05 vs. the normal group and ^#^*P* < 0.05 vs. the diabetes group).

We further investigated whether *Foxo1* overexpression could attenuate the protein indicators of apoptosis and interstitial fibrosis induced by hyperglycemia in mouse RPTCs. Although there was no change detected in the mRNA and protein levels of FOXO1 between normal and diabetic mice, the phosphorylated (p)-FOXO1/total-FOXO1 ratio was significantly higher in the diabetic conditions. Importantly, the expression level of FOXO1 increased and the p-FOXO1/total-FOXO1 ratio decreased in diabetic Tg mice. Markedly elevated expression of fibronectin (FN) and collagen IV (Col IV) was detected in RPTCs from diabetic mice compared to that in normal mice. Moreover, the expression of BAX was also enhanced in RPTCs from diabetic mice. Importantly, the expression of these indicators was significantly attenuated in *Foxo1* Tg mice. These observations demonstrated that RPTC apoptosis and interstitial fibrosis in diabetic mice can be attenuated by *Foxo1* overexpression (Figure 4).

### 3.5. Oxidative Damage Products and TXNIP-TRX Expression Are Reduced in Tg Diabetic Mice

**Figure 5:**
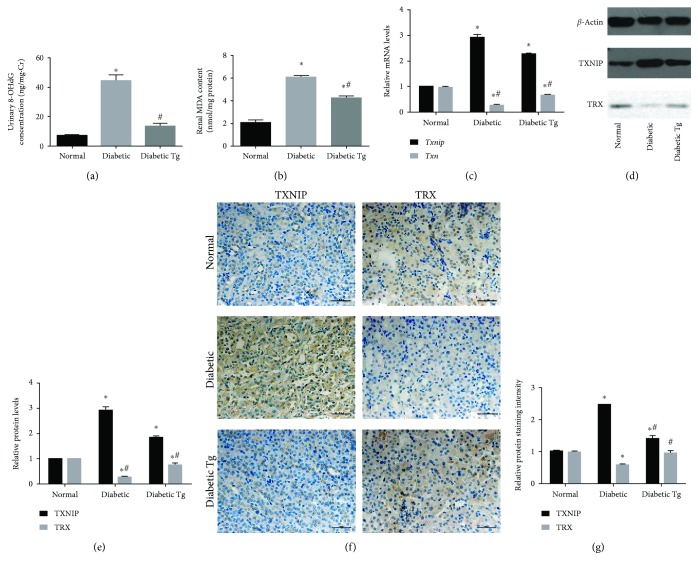
Effect of FOXO1 on oxidative damage products and TXNIP-TRX expression in diabetic mice. (a) Effect of overexpressing *Foxo1* on urinary 8-OHdG levels (ng/mg Cr) in each animal group (*n* = 9). Urinary 8-OHdG levels were measured by enzyme-linked immunosorbent assay and adjusted using urinary creatine. (b) Malondialdehyde (MDA) concentrations in the kidney tissues of various groups. (c) Relative *Txnip* and *Trx* mRNA levels measured by RT-PCR. (d, e) Protein levels of TXNIP-TRX detected by western blot analysis. (f, g) Immunohistochemistry and quantitative analysis of TXNIP and TRX expression in normal, diabetic, and transgenic (Tg) diabetic mice. Data are means ± SEM, *n* = 6 (^∗^*P* < 0.05 vs. the normal group and #*P* < 0.05 vs. the diabetic group).

Since FOXO1 plays an important role in regulating oxidative stress, we assessed the effect of *Foxo1* overexpression on oxidative stress in the kidneys of diabetic mice by detecting the expression of the DNA oxidative damage product urinary 8-oxo-2′-deoxyguanosine (8-OHdG) and the lipid peroxidation product malondialdehyde (MDA). As shown in Figures 5(a) and 5(b), the generation of urinary 8-OHDG and MDA was significantly reduced in Tg diabetic mice compared with that of diabetic mice.

Moreover, *Foxo1* overexpression significantly increased the expression level of TXNIP in the RPTCs of the diabetic mice compared to that of normal mice as determined by reverse transcription quantitative PCR (RT-PCR), western blot, and immunohistochemistry analysis. The mRNA and protein levels of TRX were also significantly reduced in diabetic mice as expected. Importantly, TXNIP expression was downregulated while TRX expression was upregulated in the RPTCs of Tg diabetic mice (Figures 5(c)–5(g)). These results demonstrated that *in vivo Foxo1* upregulation expression led to increased TRX expression accompanied by reduced oxidative stress.

### 3.6. FOXO1 Overexpression Changed the Expression of TXNIP and TRX in Human RPTCs under a High-Glucose Condition In Vitro

To verify the relationship between FOXO1 activity and TXNIP-TRX expression in RPTCs, we analyzed the expression levels of the three factors in *FOXO1* knockin (KI) and knockout (KO) human proximal epithelial tubule HK-2 cells cultured under a high-glucose (HG, 4.5 g/L glucose) condition *in vitro*.

**Figure 6:**
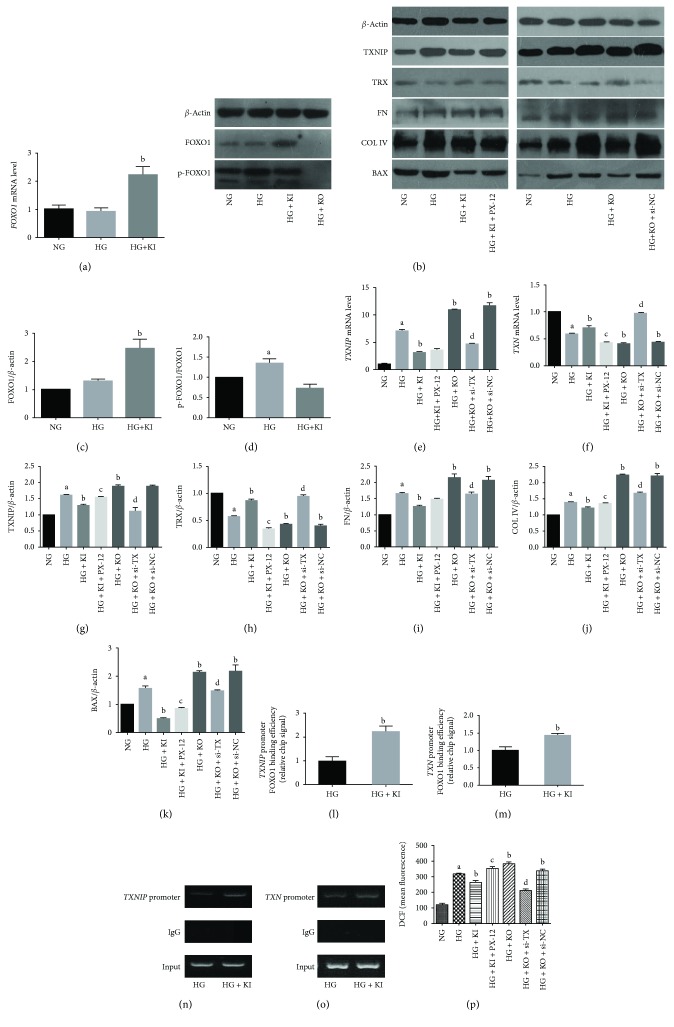
TXNIP-TRX expression in HK-2 cells cultured under high glucose. (a) Expression of FOXO1 and p-FOXO1 detected by RT-PCR. *FOXO1* knockin (KI) and knockout (KO) HK-2 cells were established with CRISPR/Cas9 and cultured under high glucose (HG; 4.5 g/L glucose) or normal glucose (NG; 1.0 g/L glucose) conditions. (b) Representative immunoblots. (c, d) FOXO1 and ratio of p-FOXO1/total FOXO1 as determined by densitometric analysis. (e–h) mRNA and protein levels of TXNIP and TRX detected by quantitative RT-PCR analysis of total RNA and western blot, respectively. *FOXO1* KI cells were treated with the TRX inhibitor PX-12, and *FOXO1* KO cells were treated with a small interfering RNA against *TXNIP* (si-TX). (i–k) The fibrosis- and apoptosis-related proteins, (i) FN, (j) COL IV, and (k) BAX, were detected by western blot analysis. (l–o) Chromatin immunoprecipitation assays showing FOXO1 binding to the promoter regions of *TXNIP* and *TXN* in HK-2 cells under HG. Soluble chromatin was immunoprecipitated with antibodies against FOXO1. The DNA fragments were analyzed by qPCR (l, m) or amplified by PCR and visualized on agarose gels (n, o). (p) Overexpression of *FOXO1* prevents reactive oxygen species (ROS) accumulation in HG-treated HK-2 cells. Intracellular ROS production was quantified by flow cytometry analysis using 2′,6′-dichlorofluorescein diacetate. The data are presented as the means ± SEM (*n* = 3). ^a^*P* < 0.05 vs. normal glucose (NG); ^b^*P* < 0.05 vs. HG; ^c^*P* < 0.05 vs. *FOXO1* knockin (KI); ^d^*P* < 0.05 vs. *FOXO1* knockout (KO).

Although there was no difference in the mRNA and protein levels of FOXO1 between the normal glucose (NG, 1 g/L) and high-glucose groups, the p-FOXO1/total-FOXO1 ratio increased significantly in the HG group compared to that of the NG group. Importantly, *FOXO1* KI increased the expression of FOXO1 and decreased the p-FOXO1/total-FoxO1 ratio in the HG-treated cells. Likewise, the mRNA and protein levels of TXNIP were significantly increased in HG-treated HK-2 cells compared with the NG group, while the TRX expression level was markedly reduced. However, the modulation of TXNIP-TRX reversed in the HG-treated KI cells. In contrast, TXNIP expression was increased, and TRX expression was decreased in the cells with *FOXO1* KO cells under the HG condition. When the HG-treated *FOXO1* KO cells were infected with *TXNIP* small interfering (si-TX), the mRNA and protein expression levels of TXNIP were reduced and the protein level of TRX was increased, whereas transfection with control siRNA (si-NC) did not induce these changes. Treatment with TRX inhibitor PX-12 also significantly reduced the protein level of TRX. Taken together, these observations indicated that *FOXO1* overexpression prevented the HG-induced upregulation of TXNIP and downregulation of TRX in cultured HK-2 cells under high glucose (Figures 6(a)–6(h)).

### 3.7. FOXO1 Binds to the TXNIP and TRX Promoters In Vitro

To determine the potential role of FOXO1 in *TXNIP* and *Txn* gene transcription in HK-2 cells, the interaction between FOXO1 and the *TXNIP* and *Txn* prompter elements was examined by chromatin immunoprecipitation (ChIP) assays, using an antibody specific for FOXO1. As shown in Figures 6(l)–6(o), FOXO1 clearly bound to the *TXNIP* and *Txn* promoter regions in cultured HK-2 cells, and FOXO1 overexpression strongly promoted these bindings.

### 3.8. FOXO1 Regulates the Accumulation of ROS via TXNIP-TRX

To verify whether FOXO1 attenuate ROS accumulation through the action of TXNIP-TRX, we measured the cellular ROS production using flow cytometry. HG-induced ROS production was decreased in *FOXO1* KI cells compared with that detected in the HG group. However, this effect was reversed by treatment with the TRX inhibitor PX-12. *FOXO1* KO increased the ROS accumulation under HG condition, whereas this effect was greatly reduced by infection with si-*TX*. There was no statistical difference in the ROS levels between the *FOXO1* KO and si-NC groups, indicating that si-NC did not affect the production of ROS. Taken together, these results demonstrated that regulating *FOXO1* alters TXNIP-TRX induced cellular ROS accumulation under high-glucose condition (Figure 6(p)).

### 3.9. Overexpression of FOXO1 Protects HK-2 Cells from HG-Induced Apoptosis and Fibrosis via TXNIP-TRX Regulation

Figures 6(b) and 6(i)–6(k) show that the protein levels of BAX, FN, and Col IV were markedly increased in the HG group compared with those of the NG group. As expected, *FOXO1* KI and KO suppressed and promoted the upregulation of these apoptoses and fibrosis indicators, respectively. In addition, the PX-12 induced downregulation of TRX expression and reversed the suppressing effect in HG-treated *FOXO1* KI cells. However, infections of HG-treated *FOXO1* KO cells with si-*TX* greatly reduced the levels of apoptosis and fibrosis indicators compared with these infected with si-NC.

**Figure 7:**
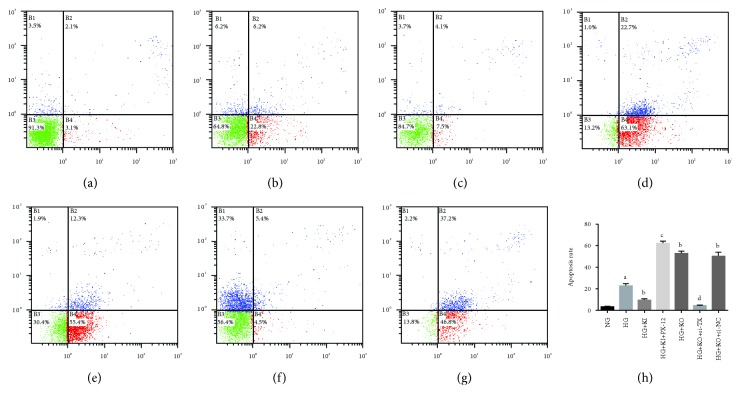
FOXO1 protects against apoptosis in HK-2 cells under high glucose via regulating TXNIP-TRX. (a–h) Apoptosis was measured by flow cytometry, using FITC-annexin V and propidium iodide as markers. Data are presented as means ± SEM (*n* = 3). ^a^*P* < 0.05 vs. normal glucose (NG); ^b^*P* < 0.05 vs. high glucose (HG); ^c^*P* < 0.05 vs. *FOXO1* knockin (KI); ^d^*P* < 0.05 vs. *FOXO1* knockout (KO).

As shown in Figure 7, the apoptosis ratio detected by flow cytometry was 3.1% in the NG group and was 22.8% in the HG group, suggesting that the HG treatment markedly increased apoptosis. In contrast, the apoptosis ratio of *FOXO1* KI cells cultured under HG was apparently reduced (7.5%), and treatment with PX-12 inhibited this protective effect of *FOXO1* overexpression. The effect of *FOXO1* KO was also largely reduced when HG-treated *FOXO1* KO cells were infected with *TXNIP* siRNA. Thus, PX-12 and *TXNIP* siRNA affect not only the expression of apoptosis-related proteins but also the overall apoptosis ratio.

## 4. Discussion

Oxidative stress plays an important role in diabetic nephropathy (DN); however, the underlying mechanism is unclear. Thioredoxin-interacting protein (TXNIP) and thioredoxin (TRX) maintain oxidative stress balance. Here, we shed new light on the regulation of TXNIP-TRX by FOXO1 in renal proximal tubular cells. *FOXO1* overexpression attenuated the high glucose-induced enhancement of TXNIP expression and impairment of TRX expression via direct binding to the promoter. Thus, by inhibiting TXNIP and promoting TRX, FOXO1 may have additional beneficial effects in protecting against oxidative damage in RPTCs, suggesting a new target for the treatment of DN.

FOXO1 regulates the expression of several genes [17] that play roles in the development and progression of diabetes mellitus [18] and DN [19]. We previously reported that forced FOXO1 activation in glomerular cells by infection with lentiviral vectors protected the mesangial cells and podocytes in the kidneys of streptozotocin-induced diabetic animals [16, 20]. However, there has been minimal research focused on the mechanisms underlying the diabetic-induced injury of the renal tubules. In this study, staining and transmission electron microscopy revealed severe tubular damage in patients with DN. Histological examinations confirmed the characteristics of renal tubular injury in diabetic mice. Moreover, *Foxo1* Tg diabetic mice (specifically overexpressing CA-*Foxo1* in the RPTCs) exhibited similar renal histology to that of normal mice, and *Foxo1* overexpression effectively attenuated the progression of albuminuria in diabetic mice. These data indicated a potentially protective role of FOXO1 in the development of DN.

As glucose-induced ROS cause injury to the podocytes and RPTCs at the onset of DN, protecting cells against oxidative stress is important. For example, overexpressing catalase in RPTCs was shown to attenuate interstitial fibrosis and tubular apoptosis in db/db mice [15]. Given the critical role of FOXO family in ROS homeostasis in diabetes mellitus [21, 22], we hypothesized that FOXO1 would be effective in inhibiting HG-induced ROS production. In support of this hypothesis, we found that oxidative stress indicators 8-OHDG and MDA were normalized in *Foxo1* Tg diabetic mice compared with those of wild-type diabetic mice *in vivo*. Similarly, overexpressing *FOXO1* attenuated the increased ROS level *in vitro* in HG-treated HK-2, while *FOXO1* downregulation increased the accumulation of cellular ROS under HG. These findings point to a relationship between FOXO1 and ROS generation, although more experiments are warranted to verify these effects and understand the mechanism.

As an important redox regulatory mechanism of intercellular ROS generation, the TXNIP-TRX interaction goes hand in hand with glomerular [23, 24] and tubular [25, 26] oxidative stress and also plays a critical role in the progression of DN. Moreover, FoxO3 was reported to reduce ROS levels by inducing TRX expression [27]. Based on these findings, we speculated that the transcription factor FOXO1 can regulate TXNIP and TRX expression in RPTCs, thereby preventing ROS-related apoptosis and fibrosis. As expected, we detected TXNIP upregulation and TRX downregulation in the RPTCs of diabetic mice, which were significantly ameliorated in Tg diabetic mice. Further supporting these effects, treatment with a TRX inhibitor and *TXNIP* siRNA could partially block the effect of *FOXO1* overexpression and KO in human RPTCs.

Nevertheless, the understanding of the mechanism(s) by which FOXO1 induces the observed changes of TXNIP and TRX remains incomplete. Previous studies have shown that FOXO regulates *TXNIP* and *TRX* transcription by bounding to their promoters and regulating their transcriptions in various cell types. For example, FOXO3 was found to bind to the *TRX* promoter and recruit the histone acetylase p300 to form a transcription activator complex in human aortic endothelial cells, resulting in a reduction in ROS levels [13, 27]. Similarly, through ChIP assay, we found a direct interaction of FOXO1 with the *TRX* promoter in HK-2 cells grown under the HG condition, and overexpression of *FOXO1* could increase the recruitment on the *TRX* promoter and prevent the HG-induced reduction of TRX expression.

TXNIP regulation is known to be tissue- and disease-specific. The FOXO family has previously been shown to differentially regulate TXNIP expression in hepatoma carcinoma cells, glucose-treated endothelial cells, and beta cells. FOXO1 bound to the *TXNIP* promoter, leading to an increase of *TXNIP* in HepG2 cells [28, 29]. However, in the normal liver, *TXNIP* expression was directly repressed by FOXO1A [30]. The specific role of FOXO1 on *TXNIP* regulation may result from the heterogeneous nature of hepatoma carcinoma cells and other proteins in the transcription complex. In beta cells, FOXO1 inhibited *TXNIP* transcription, probably by interfering with the DNA binding of ChREBP in the target gene promoters [12]. However, until now nothing has been known about the regulation of *TXNIP* by FOXO1 in renal proximal tubular cells. Our date indicated that FOXO1 bound to the *TXNIP* promoter in HK-2 treated with HG and increased the recruitment of FOXO1 on *TXNIP* promoter in *FOXO1* KI resulting in a decrease of HG-induced TXNIP enhancement, which is consistent with its effects in the beta cells and liver.

## 5. Conclusions

In summary, the present study suggested a critical role of FOXO1 in attenuating ROS production, albuminuria, RPTC apoptosis, and interstitial fibrosis both *in vivo* and *in vitro*. FoxO1 bound to the *TXNIP* and *TXN* promoter and regulates the oxidative stress balance maintained by TXNIP-TRX. These findings indicate that modulating the FOXO1/TXNIP-TRX pathway may have therapeutic utility in the treatment of DN.

## Figures and Tables

**Table 1:** Primer sequences used for quantitative reverse transcription polymerase chain reaction.

Target gene	Forward primer	Reverse primer
*ACTB* (h)	CCTGGCACCCAGCACAAT	GGGCCGGACTCGTCATAC
*ACTB* (m)	GTGCTATGTTGCTCTAGACTTCG	ATGCCACAGGATTCCATACC
*FOXO1* (h/m)	CAGCAAATCAAGTTATGGAGGA	TATCATTGTGGGGAGGAGAGTC
*TXNIP* (h/m)	CGCCACACTTACCTTGCCAATG	GCTCTTGCCACGCCATGATG
*TXN* (h/m)	TTCTCTGCTACGTGGTGTGG	AGCAACATCCTGGCAGTCAT

h, human; m, mouse.

**Table 2:** Body weight, kidney weight/body weight ratio, and biochemical indicators in the normal, diabetic (DM), and *Foxo1* transgenic diabetic (Tg DM) groups at week 12 after injection.

Group	*n*	BW (g)	KI (×10-3)	BG (mmol/L)	UTP (mg)	Scr (*μ*mol/L)	Urea (mmol/L)
NG	9	28.0 ± 0.9	5.3 ± 0.5	9.1 ± 1.1	2.0 ± 0.3	11.9 ± 1.0	9.0 ± 0.8
DM	9	22.2 ± 1.1^∗^	9.2 ± 0.3^∗^	28.5 ± 1.6^∗^	16.7 ± 0.9^∗^	28.9 ± 2.4^∗^	17.3 ± 1.2^∗^
Tg DM	9	22.2 ± 1.3^∗^	7.2 ± 0.3^∗#^	27.0 ± 2.1^∗^	12.5 ± 1.4^∗#^	20.3 ± 2.3^∗#^	13.5 ± 1.0^∗#^

The data are displayed as the means ± standard errors. BW, body weight; KI, kidney weight/body weight ratio; BG, blood glucose; UTP, 24 h urinary total protein; Scr, serum creatinine. ^∗^*P*, 0.05 vs. the normal group. ^#^*P*, 0.05 vs. the DM group.

## Data Availability

The data used to support the findings of this study are included within the article.

## Abbreviations

DCFH-DA: 2′7-Dichlorodihydrofluorescein diacetate
DN: Diabetic nephropathy
FITC: Fluorescein isothiocyanate
FN: Fibronectin
FOXO1: Forkhead transcription factor O1
HG: High glucose
KI: Knockin
KO: Knockout
MDA: Malondialdehyde
NG: Normal glucose
PBS: Phosphate-buffered saline
PI: Propidium iodide
ROS: Reactive oxygen species
RPTCs: Renal proximal tubular cells
SCr: Serum creatinine
Tg: Transgenic
TRX: Thioredoxin
TXNIP: Thioredoxin-interacting protein
UTP: Urinary total protein.
